# An Anti-Inflammatory Sterol Decreases Obesity-Related Inflammation-Induced Insulin Resistance and Metabolic Dysregulation

**DOI:** 10.1155/2013/814989

**Published:** 2013-01-30

**Authors:** Chris L. Reading, Jaime Flores-Riveros, Dwight R. Stickney, James M. Frincke

**Affiliations:** Harbor Therapeutics, Inc., San Diego, CA 92122, USA

## Abstract

Obesity-related inflammation-induced insulin resistance and metabolic dysregulation were investigated in retrospective analysis of placebo hematologic and metabolic laboratory data from trials associated with increasing chronic low-grade inflammation and body mass index. Studies included healthy subjects and those with progressive stages of metabolic dysregulation, including type 2 diabetes mellitus with uncontrolled hemoglobin A1c. Intrasubject variances in erythroid and metabolic values increased with metabolic dysregulation. Random effects were demonstrated in treatment-naïve diabetes for erythroid, glucose, and HbA1c fluctuations. The anti-inflammatory insulin sensitizer, HE3286, was tested for its ability to decrease obesity-related inflammation-induced insulin resistance and metabolic dysregulation in diabetes. HE3286 significantly decreased erythroid and metabolic variances and improved 1,5-anhydroglucitol (a surrogate of postprandial glucose) compared to the placebo group. HE3286 HbA1c decrease correlated with weight loss and inversely with baseline monocyte chemoattractant protein-1 (MCP-1) in metformin-treated diabetics. Normalization of HbA1c to the 84-day average hemoglobin revealed that HE3286 HbA1c decrease correlated with high baseline MCP-1 and MCP-1 decrease in treatment-naïve diabetics. HE3286 decreased insulin resistance, increased the frequency of decreased day 84 HbA1c in metformin-treated subjects, and decreased day 112 HbA1c in treatment-naïve diabetics. HE3286 may be useful to restore metabolic homeostasis in type 2 diabetes.

## 1. Introduction and Purpose

HE3286 (17*α*-ethynylandrost-5-ene-3*β*,7*β*,17*β*-triol) is a chemical derivative of the natural mammalian sterol androst-5-ene-3*β*,7*β*,17*β*-triol (*β*AET). *β*AET exhibits anti-inflammatory activity in rodent models, is elevated in plasma of obese subjects with normal glucose disposal, and may play a compensatory role in preventing development of metabolic syndrome (reviewed in [1]). *β*AET is pharmaceutically unsuitable, due to poor oral bioavailability and its propensity for oxidative inactivation by 17*β*-hydroxysteroid dehydrogenase [1]. HE3286 is stabilized against oxidation at position 17 and consequently orally bioavailable, does not bind to any known nuclear steroid hormone receptors, and is pharmacologically unrelated to androgens, estrogens, corticosteroids, or peroxisome proliferators [1]. HE3286 has shown broad anti-inflammatory activity in animal models of rheumatoid arthritis, ulcerative colitis, multiple sclerosis, lung inflammation, autoimmune type 1 diabetes, and neuroinflammation (reviewed in [1]). In these models, nuclear factor kappa B (NF*κ*B) activation and proinflammatory cytokine production were consistently suppressed. Furthermore, HE3286 was not markedly immunosuppressive in rodent models of ovalbumin immunization, *Klebsiella pneumoniae* or *Pseudomonas aeruginosa* infection, Coxsackievirus B3 myocarditis, delayed-type hypersensitivity, and mitogen-induced proliferation, or in the human mixed lymphocyte reaction assay (reviewed in [1]). 

Obesity induces an insulin-resistant state in adipose tissue [2], liver, and muscle and is a strong risk factor for the development of type 2 diabetes mellitus [3]. In adipose tissue, MCP-1 and tumor necrosis factor alpha (TNF*α*) play dominant proinflammatory roles [2]. Adiposity-induced inflammation-stimulated kinases phosphorylate insulin receptor substrate-1 on serine residues and inhibit insulin signaling [4]. Two recent publications report the activity of HE3286 against *in vitro* inflammatory responses and *in vivo* rodent models of obesity-induced inflammation and insulin resistance [5, 6]. HE3286 suppressed endotoxin-induced NF*κ*B activation, reporter gene expression, nuclear localization, and p65 phosphorylation in mouse macrophages and decreased phosphorylation of the proinflammatory extracellular signal-regulated (Erk1/2), IkappaB (Ikk), Jun N-terminal (Jnk), and p38 mitogen-activated protein (p38 Mapk) kinases. HE3286 also attenuated TNF*α*-stimulated inflammation and TNF*α*-induced adipocyte-stimulated macrophage chemotaxis [5, 6]. HE3286 treatment of diabetic *db/db* mice, insulin-resistant diet-induced obese mice, and genetically obese *ob/ob* mice suppressed progression to hyperglycemia and markedly improved glucose clearance. This effect appeared to be consequent to reduced insulin resistance, since HE3286 lowered blood insulin levels in both *db/db* and *ob/ob* mice. In these studies HE3286 suppressed levels of the chemokine monocyte chemoattractant protein-1 (MCP-1), along with its cognate receptor, C-C motif chemokine receptor-2, in white adipose tissue [6]. In Zucker diabetic fatty rats, HE3286 downregulated inflammatory cytokine and chemokine expression in both liver and adipose tissues and suppressed macrophage migration into adipose tissue. HE3286 normalized fasting and fed glucose levels, improved glucose tolerance, and enhanced skeletal muscle and liver insulin sensitivity, as assessed by hyperinsulinemic, euglycemic clamp studies. In addition, HE3286 reduced liver cholesterol and triglyceride content, leading to a feedback elevation of low-density lipoprotein (LDL) receptor and decreased total serum cholesterol [5]. Recently, we have reported that HE3286 binds to Erk1/2, Lrp1, and Sirt2 [7] and proposed that the HE3286-mediated decrease in hyperactivation of Erk1/2 may be causal for its metabolic [8] and anti-inflammatory activities. 

In a clinical study in obese, impaired glucose tolerance (IGT) subjects, HE3286 significantly increased the frequency of insulin-resistant subjects with improved day 29 insulin-stimulated glucose disposal, increased HDL cholesterol, and decreased day 28 CRP compared to placebo-treated subjects [9]. Based on baseline glucose clamp studies, insulin-resistant subjects had elevated inflammatory biomarkers, with lower adiponectin and higher cytokine secretion in LPS-stimulated PBMC. After 28 days of HE3286 treatment, adiponectin levels increased significantly in insulin-resistant subjects, compared to placebo. These results support our hypothesis that obesity-induced inflammation is a significant contributor to metabolic dysregulation and that the anti-inflammatory activity of HE3286 can preferentially benefit the insulin-resistant inflamed subpopulation of obese IGT subjects. 

Based on preclinical studies and these foregoing results in IGT subjects, it was conjected that HE3286 might benefit obese inflamed insulin-resistant individuals with type 2 diabetes mellitus (T2DM). A widely accepted clinical endpoint for T2DM is the change in HbA1c, a surrogate marker for the extent of hyperglycemia an individual experiences over time. Traditionally, erythroid hematology values are considered stable in healthy individuals, and hemoglobin and HbA1c turnover is reported to reflect the normal red cell half-life of 38–60 days [10]. In T2DM, the life span of red cells can be altered significantly by inflammation, particularly TNF*α*-induced oxidative stress [11], obese low-grade systemic inflammatory response syndrome [12], the presence of elevated levels of advanced glycation endproducts on the surface of red cells [13, 14], hypoxia [15], and excessive erythrocytosis [16]. There are reports of large fluctuations in HbA1c in type 1 diabetes [17], especially in subjects with poor glycemic control [18, 19]. This information prompted us to also assess the association of obesity-related chronic low-grade inflammation with hemoglobin concentration and HbA1c variability in uncontrolled T2DM. We retrospectively analyzed the hematologic and metabolic clinical laboratory data for placebo groups from 10 clinical studies that were conducted between 2001 and 2010. These studies included both healthy subjects and individuals in progressive stages of metabolic disease that presented with increased chronic low-grade inflammation coincident with elevated BMI that included dyslipidemic, IGT, and T2DM participants with uncontrolled HbA1c. 

With an understanding of the variability associated with progressive adiposity, inflammation, and metabolic disease, we assessed the activity of HE3286 to decrease obesity-induced inflammation and insulin resistance in T2DM. 

## 2. Subjects and Methods

### 2.1. Studies

This paper reports the activity of HE3286 in T2DM patients with uncontrolled HbA1c. High metabolic and hematologic laboratory value variances were observed in these patients. For comparison, similar parameters were retrospectively analyzed from placebo subjects enrolled in 10 clinical studies conducted by Harbor Therapeutics, Inc. (formerly Hollis-Eden Pharmaceuticals, Inc.) since 2001. These studies included healthy volunteers, dyslipidemic, IGT, and T2DM participants. Only placebo subjects from these studies were used for intercomparison. All studies excluded patients with known liver disease and alcoholism. The protocols and all amendments were reviewed and approved by the relevant institutional review boards, and all studies were conducted in accordance with the Declaration of Helsinki and the International Conference on Harmonization/WHO Good Clinical Practice Standards. Experimental studies were conducted with the understanding and informed consent of human subjects.

Details of studies 2100-200, -201, -202, and -203 have been published [20]. These four double-blind, randomized, placebo-controlled, healthy human safety studies were conducted in The Netherlands (Kendle International, Utrecht) and the United States (Parexel International, Baltimore, MD). Two single-dose, dose-escalation studies assured safety and evaluated the pharmacokinetics of androst-5-ene-3*β*,17*β*-diol (HE2100) (studies 200 and 202). A multidose, dose escalation study was performed to assess safety and pharmacokinetics and potential early activity of HE2100 (study 201). Early activity, defined by effects on peripheral blood elements, was confirmed by a follow-up study that included elderly subjects and an initial study of bone marrow hematology (study 203).

Details of studies 2200-100, -101, -120, and -130 have also been published [21]. Healthy adult and elderly subjects were randomized to receive three consecutive daily subcutaneous injections of placebo, 50, or 100 mg androst-5-ene-3*β*,7*β*,17*β*-triol (HE2200), followed by 2 months of periodic observation (trial 2200-100), or to receive placebo, 25, or 100 mg HE2200 transmucosally (buccal administration) once daily for five days followed by 2 months of periodic observation (trial 2200-101). Study 2200-120 was a phase II study in healthy hepatitis B-naïve, and elderly (65–85 years old) volunteers, who received hepatitis B vaccine, were randomized to concomitantly receive either 100 mg of HE2200 or placebo equivalent. Subjects received three subcutaneous injections of study drug or placebo prior to the first and second doses of hepatitis B vaccine given 28 days apart. The third dose of vaccine was given at 6 months without HE2200 or placebo treatment, and the study terminated 28 days later. 

Study 2200-130 was a phase II study in dyslipidemic subjects, ages 18–70 years, with plasma triglyceride concentrations 1.7–2 mmol/L, total cholesterol levels of 5.7–8.3 mmol/L, and HDL levels of ≤1.2 mmol/L for males and ≤1.4 mmol/L for females. After informed consent was obtained, subjects initiated a Step II AHA diet and discontinued all lipid lowering agents for a six-week run-in period. Each subject's lipid profile at week four of the diet was used to determine eligibility for the study. At six weeks, qualified subjects were randomized to receive 25 or 100 mg of HE2200 or placebo equivalent by buccal administration for 28 days.

HE3286-0102 was a multicenter, double-blind, dose-ranging phase I study designed with 5 cohorts of obese, impaired glucose tolerance (IGT), but otherwise healthy participants [9]. Subjects were screened for fasting blood glucose level of <7.0 mmol/L and a 7.8–11.1 mmol/L 2-hour postprandial glucose following a 75-gram oral glucose load. Oral placebo or HE3286 doses of 4 (2 BID), 5 (QD), 10 (5 BID), and 20 (10 BID) mg were administered daily for 28 days. One-step hyperinsulinemic, euglycemic clamps were performed on the day before the first dose and day 29 in the BID dose groups. 

For comparisons, the number of placebo subjects was 70 healthy, 24 dyslipidemic, 13 IGT, 28 treatment-naïve, and 38 metformin-treated T2DM. 

### 2.2. Placebo Data Analysis

Placebo data from 10 clinical studies was analyzed to obtain coefficients of variation (CV) for red blood cells (RBC), hematocrit (Hct), mean corpuscular volume (MCV), mean corpuscular hemoglobin (MCH), mean corpuscular hemoglobin concentration (MCHC), hemoglobin (Hb), hemoglobin A1c (HbA1c), platelets, lymphocytes, monocytes, white blood cells, fasting glucose, insulin, triglycerides, cholesterol, high-density lipoprotein cholesterol (HDL), and low-density lipoprotein cholesterol (LDL). CVs from each medical condition were compared to those of healthy volunteers for significant differences in magnitude using Welch ANOVA, allowing for unequal differences, and for significant differences in dispersion, using the 2-sided *F* test. Only placebo values were used to avoid any treatment effects from drug administrations. Pearson correlations between individual subject CVs for each parameter and CVs for HbA1c and for insulin were determined to assess commonality in increased variances between parameters. In addition, intravisit changes in HbA1c were determined for each subject with HbA1c data.

### 2.3. HE3286-0401 Data Analysis

Data for all placebo- and HE3286-treated T2DM subjects were analyzed for the same parameters by cohort (metformin treated and treatment naïve) and in subgroups (stratified by baseline monocyte chemoattractant protein-1 (MCP-1) in metformin-treated subjects, and by body mass index (BMI) in the MCP-1 selected treatment-naïve subjects). Baseline characteristics were assessed for balance between groups. 

Based on the findings of excessive variability in T2DM, including random effects in intravisit Hb, HbA1c values were normalized (nHbA1c) by the 84 day average Hb value for each subject. This was done based on the fact that random effects have a mean of zero, but an excessive dispersion. Thus the average change in placebo subjects over 84 days would be expected to be zero, and true changes in HbA1c due to treatment could be compared.

Correlations were determined for independent variables of baseline MCP-1, baseline tumor necrosis factor alpha (TNF*α*), BMI, Hb CV, and day 84 changes in homeostatic model assessment of beta cell function (HOMA2 %B), glucose, weight, Hb, TNF*α*, and MCP-1 with dependent variables Hb CV, HbA1c CV, and changes in HbA1c and 84 day average normalized HbA1c (ΔnHbA1c), in order to understand clinical parameters affecting variability and treatment effects. The magnitude of HE3286 treatment effects compared to placebo was tested in the subgroups of MCP-1 > 40 pg/mL in metformin-treated subjects, and of BMI > 31 in treatment-naïve subjects.

Heteroscedasticity (differences in variances between subgroups) was tested for changes in insulin, C-peptide, fasting glucose, HOMA2 %B and HOMA2 insulin resistance (HOMA2 IR), leptin, HbA1c, insulin, MCP-1, and triglycerides. Subgroup distributions were tested for normality (Shapiro-Wilks *W* test) for HE3286 and placebo treatment. Differences in dispersions between HE3286 and placebo treatment were analyzed using the 2-sided *F* test. 

### 2.4. Test Article HE3286

HE3286, 17*α*-ethynylandrost-5-ene-3*β*,7*β*,17*β*-triol active pharmaceutical ingredient was manufactured by Norac, Azuza, CA, and formulated and filled in gelatin capsules by Eminent Services Corporation, Frederick, MD. All manufacturing procedures were performed according to current good manufacturing practices. 

### 2.5. Study HE32866-0401

**Figure 1:**
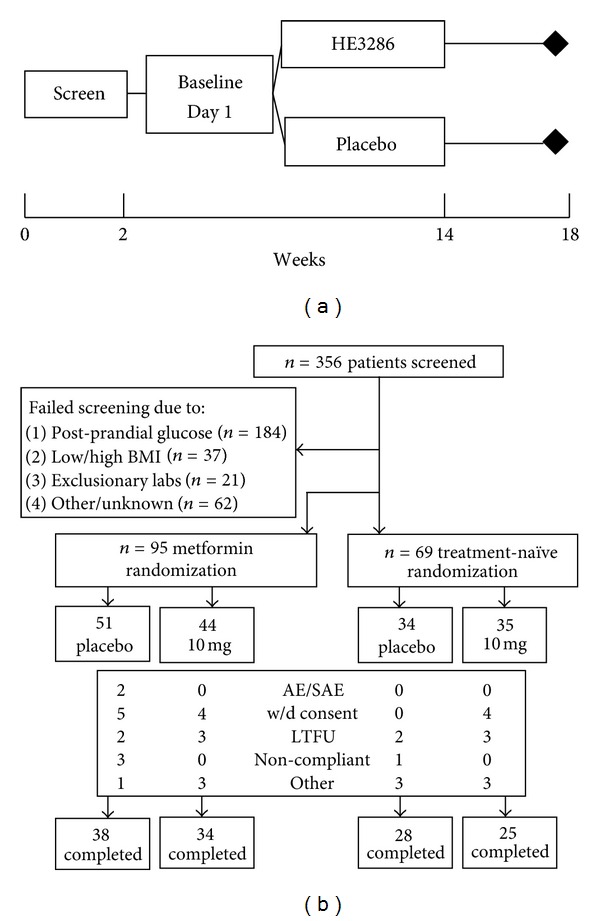
Study HE3286-0401 in type 2 diabetic subjects. HE3286-0401 study design (a) and study flow and numbers (b) for each cohort. BMI: body mass index, w/d: withdrew, and LTFU: lost to followup.

The phase II trial design was a double-blind, randomized, placebo-controlled parallel group study of the safety, tolerance, and activity of HE3286 when administered orally for 12 weeks to adult T2DM patients (Figure 1(a)). This was an adaptive design to investigate the characteristics of T2DM subjects that respond to HE3286. In cohort 1 of the study, 95 eligible patients, who consented to participate, were randomized 1 : 1 to receive study treatment (HE3286 10 mg/day or placebo) in addition to a stable dose of metformin. Inclusion criteria for cohort 1 included HbA1c ≥ 7.5% and fasting glucose ≤ 12.5 mmol/L. In cohort 2, 69 subjects who consented to participate and who met a revised eligibility criteria as determined by cohort 1 were randomized 1 : 1 to receive study treatment (HE3286 10 mg/day or placebo) as monotherapy. After the analysis of data from the first stage of the study, the population for cohort 2 was phenotypically enriched by screening for the following: HbA1c 7.0–10.5%, fasting glucose ≤12.5 mmol/L, BMI ≥ 28 kg/m^2^, insulin ≥ 27.8 pmol/L, C-peptide ≥ 0.67 nmol/L, and serum MCP-1 ≥ 36 pmol/L. Subjects were screened and enrolled through outpatient clinics. The sponsor selected sites after a site visit to determine site qualifications and the investigator's ability to conduct clinical investigations according to the protocol and current Good Clinical Practice regulations: clinical trial registration: HE3286-0401 NCT00694057 http://www.clinicaltrials.gov.

### 2.6. Analysis of Variance in Erythroid and Metabolic Parameters in Placebo Comparison Studies

Variance in erythroid parameters was examined by three ways. First, the variances for selected hematologic and metabolic laboratory values, such as the mean coefficient of variation (CV) and the CV range for each individual subject, were determined and compared with those of healthy subjects. Second, the intravisit changes in HbA1c were compared for individual subjects for each condition with those of healthy subjects. Third, intravisit and day 84 changes in HbA1c and other hematology and laboratory parameters were tested for random effects.

### 2.7. Statistical Analyses

Random effects were tested using Residual Maximum Likelihood (REML) using StatXact, and outliers were examined using Mahalanobis distance (Cytel Software Corporation, Cambridge, MA) in conjunction with SAS software (SAS Institute, Cary, NC). Correlations were tested using Spearman or Pearson correlations, and the hypothesis that placebo participants with clinical conditions have higher frequencies of abnormal hematology and laboratory values than healthy subjects was tested using one-tailed Fisher's exact test. Heteroscedasticity (tests of different variabilities between subpopulations) was tested for normal distributions (Shapiro-Wilks *W* test), and dispersion was tested using the 2-sided *F* test (Prism Graph Pad, San Diego, CA). If there were significant differences in variances between groups, they were further examined using a *t*-test assuming unequal variances, nonparametric Mann-Whitney test, or Fisher's exact test. Due to the exploratory nature of this hypothesis-testing study, *P* values were not adjusted for multiple comparisons. 

### 2.8. Normalization of HbA1c to Remove Random Effects in HE3286-0401

Through the course of this analysis it was discovered that the inflammatory status of the selected patient population created large and rapid changes in the patient's red cell mass that affected the whole body hemoglobin mass and consequently the fidelity of the HbA1c metric. In order to investigate HE3286 treatment effects on HbA1c in T2DM patients, HbA1c changes were normalized to the day 84 average Hb for each subject, by averaging Hb values acquired at each clinic visit. This is statistically justified based on the fact that random effects have a mean of zero but are characterized with high variances. Normalized HbA1c (nHbA1c) was applied to correct for the inflammation-induced variances found in this T2DM study population with uncontrolled inflammation.

### 2.9. Details of Normalization of HbA1c Using 84-Day Average Hemoglobin in HE3286-0401

**HbA1c** is reported in units of % hemoglobin (%Hb). **Hb** is the concentration of hemoglobin (reported in units of g/dL). **blood volume** (male) = 0.6041 + 0.3669 ∗ (height in meters)^3^ + 0.03219 ∗ (weight in kg). **blood volume** (female) = 0.1833 + 0.3561 ∗ (height in meters)^3^ + 0.03308 ∗ (weight in kg). (Blood volume unit = L). **total  Hb  mass** = **Hb**∗10∗** blood volume**(Hb mass units = g). **total  HbA1c = HbA1c**∗**total  Hb  mass** (total HbA1c units = g). **84 day average total  Hb  mass** = mean of baseline to day 84 **total  Hb  mass** measurements.

Normalized HbA1c (**nHbA1c**) = 100 ∗ (**total  HbA1c**/**84 day average total Hb mass**)(nHbA1c units = %Hb).

Δ**nHbA1c** represents change in **nHbA1c**.

**day 84**  Δ**nHbA1c** = **day 84 nHbA1c**−baseline **nHbA1c**.

## 3. Results

### 3.1. Retrospective Exploration of Increased Variance with Metabolic Disease Progression

The hypothesis that chronic low-grade inflammation leads to increased variance in laboratory values was explored by a retrospective review of hematology and metabolic clinical parameters from placebo subjects enrolled in 10 clinical studies conducted by Harbor Therapeutics, Inc., since 2001. Only placebo subject data from these studies were used for intercomparisons to exclude study drug effects. 

### 3.2. Variances of Hematology and Laboratory Values between Medical Conditions in Placebo Subjects

**Figure 2:**
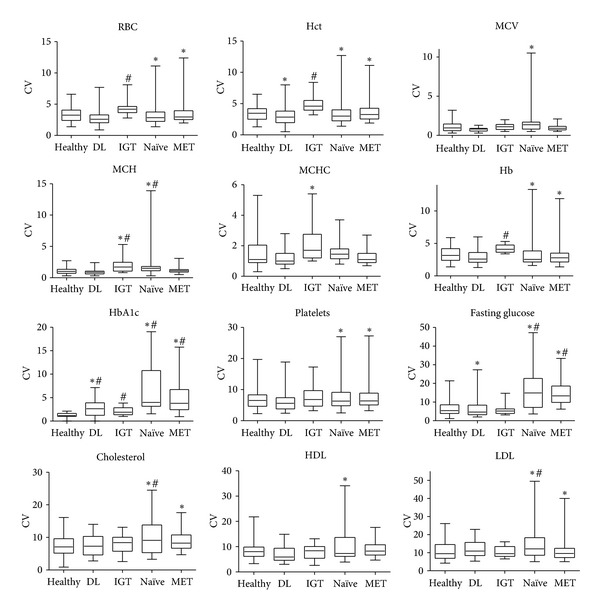
Coefficients of variation for individual subject hematology and chemistry values in placebo comparisons. DL: dyslipidemic placebo group, IGT: impaired glucose tolerant placebo group, Naïve: treatment-naïve uncontrolled T2DM placebo group, MET: uncontrolled T2DM placebo group on a stable dose of metformin, Hb: hemoglobin, Hct: hematocrit, RBC: red blood cell count, MCV: mean cell volume, MCH: mean corpuscular hemoglobin, MCHC: mean corpuscular hemoglobin concentration, HbA1c: hemoglobin A1c, HDL: high density lipoprotein cholesterol, LDL: low density lipoprotein cholesterol. ^#^Statistically significant (Welch ANOVA, allowing unequal variance) increase in mean coefficient of variation compared to healthy participants. *Statistically significant dispersion (2-sided *F* test) in the coefficient of variation compared to healthy participants.

Changes in variance (CV means and ranges) for hematologic and metabolic parameters sorted by medical condition are displayed in Figure 2. Dyslipidemic patients showed increased variances in hematocrit, HbA1c, and fasting glucose compared to healthy subjects. Although their lipid parameters were abnormal, their lipid variances were not significantly higher than those of healthy subjects. IGT subjects had significantly higher variances for RBC, hematocrit, mean corpuscular hemoglobin, mean corpuscular hemoglobin concentration, hemoglobin, and HbA1c. Although they had higher postprandial glucose, their fasting glucose variances were not significantly greater than those of healthy subjects. Metformin-treated T2DM patients had significantly higher variances for RBC, hematocrit, hemoglobin, HbA1c, platelets, fasting glucose, cholesterol, and LDL. Treatment-naïve T2DM patients had significantly elevated variances for RBC, hematocrit, mean cell volume, mean corpuscular hemoglobin, hemoglobin, HbA1c, platelets, fasting glucose, cholesterol, HDL and LDL. 

### 3.3. Intravisit HbA1c Changes in Placebo Subjects

**Figure 3:**
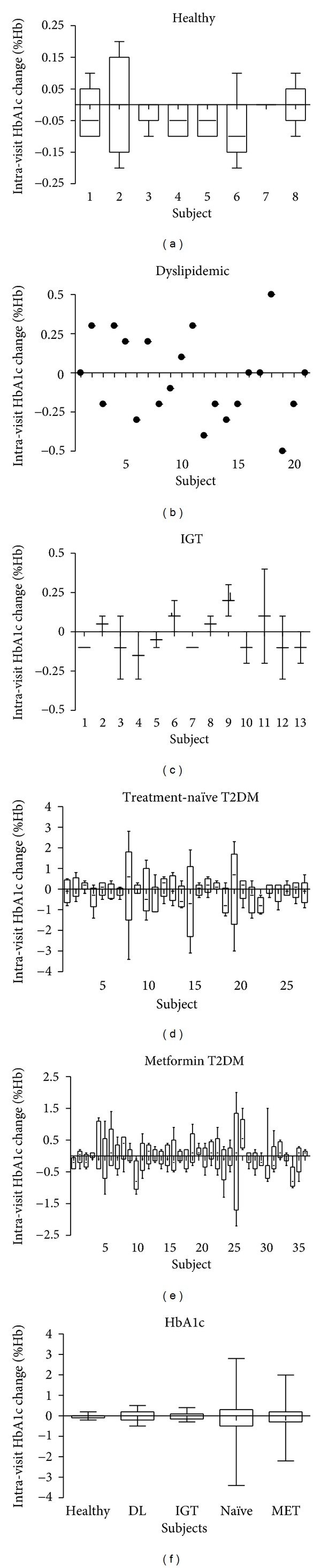
Intrasubject HbA1c changes in placebo comparisons. Bar and whisker plots of the change in HbA1c between visits for individual subjects: (a) healthy subjects from study 2100-202, (b) dyslipidemic subjects from study 2200-130, (c) impaired glucose tolerance subjects from study 3286-0102, (d) treatment-naïve type 2 diabetes patients from HE3286-0401 cohort 2, (e) type 2 diabetic patients on a stable dose of metformin from HE3286-0401 cohort 1, (f) intravisit changes for all subjects from each condition plotted on the same scale. IGT: impaired glucose tolerant, Metformin T2DM: uncontrolled type 2 diabetes mellitus participants on a stable dose of metformin.

Individual subject intravisit HbA1c changes are presented by medical condition in Figure 3. Healthy subjects HbA1c values were only collected in study 2100-202. Over 28 days, the 8 subjects showed an intravisit median change of 0 and a range from −0.2 to 0.2% Hb, consistent with literature reports. HbA1c was measured in dyslipidemic patients on days 1 and 28, yielding a single intravisit value for 21 patients with a median of 0 and an increased range of −0.5 to 0.5% Hb. Subjects with dysregulated glucose showed a median change of 0 with increased ranges: IGT over 56 days (−0.3 to 0.4% Hb), metformin T2DM over 112 days (−2.2 to 2.0% Hb), and treatment-naïve T2DM over 112 days (−3.4 to 2.8% Hb).  Figure 3(f) shows the intravisit changes in HbA1c for each medical condition on the same scale. Intravisit HbA1c changes for individual T2DM patients showed 10-fold increases over healthy subjects. These results indicate that the intravisit variances for individual T2DM patients are increased and distinct from the normal variances in healthy subjects.

### 3.4. Correlations with HbA1c and Insulin Variance in Treatment-Naïve Placebo Subjects

Treatment-naïve T2DM patients had the greatest variance in HbA1c. In this group, Pearson correlations were used to investigate the HbA1c and insulin variance relationships with other clinical parameter variances (Table 1). Individual patient HbA1c CV, were correlated with CVs for hemoglobin, MCP-1, glucose, CRP, HDL, LDL, triglycerides, lymphocytes, monocytes, platelets, RBC, hematocrit, and MCV, and insulin CVs were similarly correlated with CVs for glucose, WBC, and neutrophils, indicating dysregulation of multiple hematopoietic and metabolic functions within the same individual. Furthermore, REML analyses demonstrated significant random changes in day 84 glucose (*P* < 0.0001), hemoglobin (*P* < 0.0001), HbA1c (*P* = 0.02), and HOMA2 %B (*P* = 0.004), indicating laboratory results unreflective of the clinical situation. 

### 3.5. Conclusions from the Retrospective Analysis of Placebo Subjects with Medical Conditions

There appears to be a progressive increase in metabolic and hematologic laboratory parameter variances with increased BMI and metabolic disease progression that results in random HbA1c changes. Based on this, intervention with the HE3286 anti-inflammatory agent might confer benefit to this pathology. However, with random HbA1c effects in the placebo participants, it is difficult to demonstrate significant changes with comparisons to active agents unless a correction is applied to the data. Accordingly, treatment effects were investigated by normalizing HbA1c to the day 84 average total body hemoglobin mass for each patient.

### 3.6. HE3286-0401

In this study, HE3286 was well tolerated. Seventy-six percent of cohort 1 (metformin treated) and 77% of cohort 2 (treatment naïve) completed the study (Figure 1(b)). Only 1 serious adverse event occurred, a transient asymptomatic elevation from baseline of blood amylase, which resolved on study. This event was considered by the investigator to be possibly related to study medication. There were no clinically significant abnormalities related to any body system, including hypoglycemia and electrocardiograms, attributable to HE3286 administration. There were no detectable differences or trends in adverse events between placebo- and HE3286-treated subjects. No patient died while on study. Baseline demographics and characteristics of each group are presented in Table 2.

### 3.7. Correlates of HbA1c and Hemoglobin Changes and Variances in HE3286 and Placebo Subjects

#### 3.7.1. Cohort 1

Correlations of ΔHbA1c, HbA1c CV, and Hb CV with other parameters are shown in Table 3. The HE3286 cohort 1 group HbA1c change was negatively correlated with baseline MCP-1 (*P* = 0.01) and change in HOMA2 %B (*P* = 0.01) and positively correlated with glucose change (*P* = 0.009), weight change (*P* = 0.007), and change in Hb (*P* = 0.03). HbA1c change was positively correlated with fasting glucose change in both HE3286 and placebo. The placebo HbA1c change was negatively correlated with baseline TNF*α* (*P* = 0.004) and positively with change in TNF*α* (*P* = 0.02). In addition, in the placebo group, intrapatient HbA1c coefficients of variation (CV) were significantly correlated with baseline TNF*α* (*P* = 0.002). These relationships led to the hypothesis that HE3286 decreased HbA1c in the more inflamed (higher MCP-1) patients, in conjunction with increased pancreatic beta cell function and weight loss, but in placebos, inflammation (TNF*α*) was primarily contributing to HbA1c changes. The finding that Hb change was positively correlated with HbA1c change suggested the possibility that inflammation-induced effects produce random Hb levels that contribute to increased variance in HbA1c changes. 

High intrapatient CVs were observed for both HbA1c (up to 16%) and Hb (up to 12%) in both HE3286 and placebo groups (data not shown). Residual maximum likelihood (REML) analyses indicated a significant random CV component in the intrapatient Hb values, for both the HE3286 and placebo groups (*P* < 0.0001 for each, data not shown).

#### 3.7.2. Cohort 2

 Cohort 2 participants were selected using more stringent criteria for MCP-1, BMI, insulin, and C-peptide. TNF*α* was not measured in this group. Consequently, significant correlations observed in cohort 1 for HbA1c change with baseline MCP-1 and changes in weight and in TNF*α* were not observed in the overall cohort 2 group. Table 3 also shows correlations between ΔHbA1c, HbA1c CV, and Hb CV and other parameters in cohort 2. In this overweight to obese population, ΔHbA1c was correlated negatively with baseline BMI (*P* = 0.04) and with HOMA2 %B change (*P* = 0.02) and positively with day 84 change in fasting plasma glucose (*P* = 0.001) for HE3286, but not placebo. Further, we found that the cohort 2 participants (selected for higher inflammation) had higher variances in erythroid hematology values than cohort 1 (see Figure 2), and cohort 2 placebo day 84 changes in HbA1c had a significant random component (REML *P* = 0.006, data not shown). In placebo patients, HbA1c CV was positively correlated with baseline Hb CV (*P* = 0.02) and the ΔMCP-1 inflammation marker (*P* = 0.0495) and negatively correlated with weight change (*P* = 0.02). Hb CV, in turn, was positively correlated with baseline MCP-1 (*P* = 0.04). These relationships led us to the hypothesis that HE3286 decreased HbA1c in patients with higher obesity (BMI), in conjunction with improved pancreatic beta cell function and decreased fasting glucose, that, for placebo patients, HbA1c change and Hb CV were related to inflammation status (MCP-1), and that weight loss in placebos might be related to inflammation effects on malnutrition. 

Together, the results from studies in HE3286-0401 T2DM patients suggested that low-grade chronic inflammation develops during metabolic disease progression in the obese diabetic and contributes to dysregulation of metabolic and hematologic homeostasis. If this is correct, then intervention with an anti-inflammatory compound such as HE3286 might lead to restoration of homeostasis, normalization of glucose levels, and a decline in weight. Further, the inflammatory effects on erythropoiesis may be quelled and the utility of ΔHbA1c as a biomarker of glucose control restored. 

### 3.8. HE3286-0401 Treatment Effects

#### 3.8.1. Cohort 1

According to our observations in phase I inflamed obese prediabetics, HE3286 should show benefit in inflamed T2DM individuals. The correlation of baseline MCP-1 in the HE3286 HbA1c response was explored, and significant treatment effects were observed in the more inflamed subjects (baseline serum MCP-1 upper 2 tertiles (>40 pmol/L)).  Table 4 displays the HE3286 day 84 treatment effects on clinical parameters in this subgroup. Significant decreases were observed for HOMA2 IR (*P* = 0.02), C-peptide (*P* = 0.04), Hb (*P* = 0.02), Hct (*P* = 0.02), and RBC (*P* = 0.02) changes in the HE3286 treatment group when compared to the placebo (metformin alone) group.

The effect of HE3286 on ΔnHbA1c in the overall population was not significant. Therefore, the day 84 treatment effect on ΔnHbA1c was investigated in the more inflamed MCP-1 subgroup (Table 4). The median magnitude of the ΔnHbA1c was −0.44% Hb (HE3286, −0.34; placebo, +0.1). The HE3286 data was normally distributed, but placebo was significantly abnormal (*P* = 0.0006, *W* test, data not shown). This situation necessitated the use of nonparametric methods of data analysis. The HE3286 treatment effect was found to significantly decrease nHbA1c from zero (*P* = 0.03). The frequency of HE3286 patients with decreased nHbA1c was significantly greater than placebo (17/22 versus 9/25, *P* = 0.0008). There were no significant differences between HE3286 and placebo groups at follow-up day 112. 

**Figure 4:**
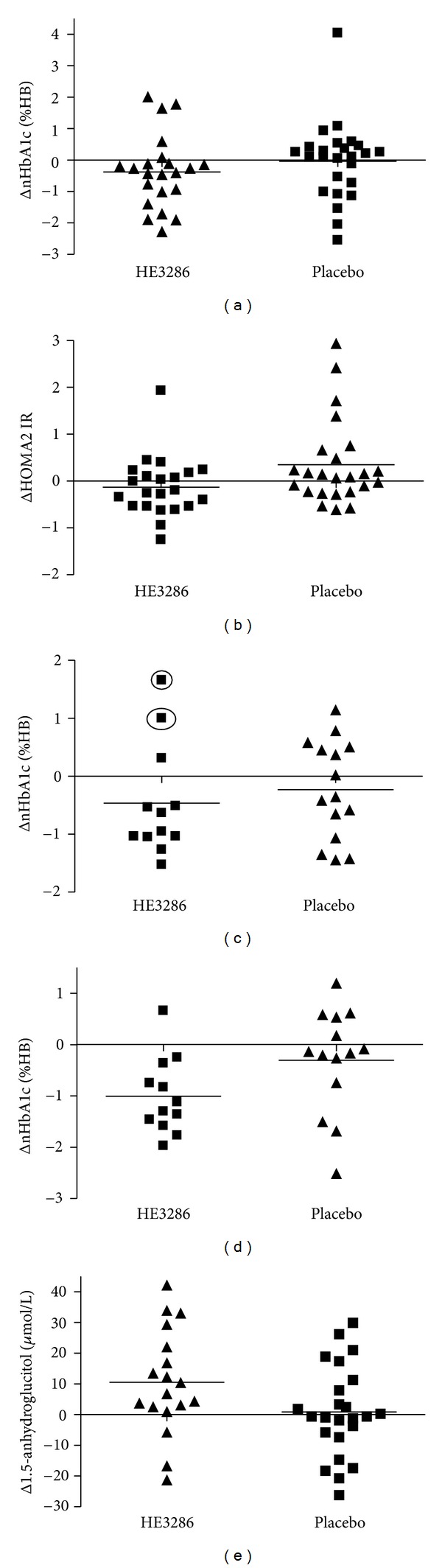
HE3286-0401 distributions of changes by participant. (a) Day 84 cohort 1 baseline MCP-1 > 40 pmol/L individual subject changes in normalized HbA1c by treatment. (b) Day 84 cohort 1 baseline MCP-1 > 40 pmol/L individual subject changes in homeostatic model assessment of insulin resistance by treatment. (c) Day 84 cohort 2 baseline BMI > 31 kg/m^2^ individual subject changes in normalized HbA1c by treatment. The circles indicate two outliers detected using Mahalanobis distance. (d) Day 112 cohort 2 baseline BMI > 31 kg/m^2^ individual subject changes in normalized HbA1c by treatment. (e) Pooled 1,5 anhydroglucitol changes from day 84 cohort 1 and 2 analyses by treatment. Analyses were performed on a subset of patients with available retention samples, predominantly those in cohort 1 with baseline MCP-1 > 40 pmol/L and in cohort 2 with baseline BMI > 31 kg/m^2^.

The HE3286 and placebo patients distributions with MCP-1 > 40 pmol/L are shown for ΔnHbA1c in Figure 4(a) and for ΔHOMA2 IR in Figure 4(b). The majority of HE3286 patients showed decreased nHbA1c and HOMA2 IR, whereas the majority of placebos showed increases. These results are consistent with inhibition of NF*κ*B hyperactivation and consequent restoration of normal insulin signaling, consistent with the preclinical HE3286 observations. 

#### 3.8.2. Cohort 2

The correlation between baseline BMI and change in HbA1c in the HE3286 group was explored by stratifying participants on the median BMI (31 kg/m^2^). The ΔnHbA1c in the HE3286, but not placebo participants (with BMI > 31 kg/m^2^), correlated significantly with their baseline MCP-1 (*P* = 0.03) (Table 3). This strengthens the hypothesis that HE3286 benefited the obese inflamed subset of T2DM patients. The ΔnHbA1c also correlated significantly with ΔMCP-1 (*P* = 0.002) in HE3286 (Table 3), but not in placebo participants. Thus the decrease in inflammation (MCP-1) was associated with the decrease in HbA1c with HE3286 treatment. 

The effect of HE3286 on nHbA1c in the overall population was not significant. The obese patients with a BMI (>31 kg/m^2^), demonstrated a significant treatment effect (*t*-test) to decrease nHbA1c by 0.6% Hb compared to placebo, but only after exclusion of 2 outliers (Mahalanobis distance). The day 84 distribution of the ΔnHbA1c for BMI > 31 is shown in Figure 4(c) (outliers circled). The variances were much higher in the treatment-naïve patients' parameters compared to uncontrolled metformin-treated patients (Figure 3(f)). We speculated that these two outliers were still subject to inflammation-induced random effects, after only 84 days of treatment and that additional treatment may be necessary to observe effects in these individuals. Because of the lag in ΔHbA1c following glucose excursions, we tested the treatment effects on follow-up day 112. A significant day 112 treatment effect (with no outliers) was found in the high BMI stratum, both by nonparametric and parametric tests (Table 4). HE3286 participants had a significant mean change from baseline (−1.0% Hb, *P* = 0.0007), whereas placebo did not. The mean change compared to placebo was also significant (−0.7% Hb, *P* = 0.03). The HE3286 participants also had a significant median change from baseline (−1.2% Hb, *P* = 0.002), whereas placebo did not. The magnitude of the response in the HE3286 treatment groups was significant (−1.0% Hb, *P* = 0.02) compared to placebo, as was the frequency of subjects with a 0.5% HbA1c decrease (9/12 versus 4/13, *P* < 0.05). The day 112 ΔnHbA1c distributions for BMI > 31 kg/m^2^ are shown in Figure 4(d).

### 3.9. Postprandial Treatment Effect

A significant treatment effect that lowered fasting glucose was not found and was attributed to high metabolic parameter variations. Consequently, the possibility that HE3286 decreased HbA1c through action on postprandial glucose was investigated. Serum 1,5-anhydroglucitol (1,5-AH) is a dietary human metabolite that is reabsorbed by a kidney glucose transporter [22]. The 1,5-AH level declines when blood glucose levels are elevated above 10 mmol/L and likewise increases when the blood glucose level declines. 

1,5-AH was measured in a subset of 42 participants (19 from stages 1 and 23 from stage 2) that had available day 84 retention samples. Analysis of 18 patients treated with HE3286 demonstrated that their 1,5-AH concentration increased significantly (+10.4 *μ*mol/L, *P* = 0.02); 24 treated with placebo demonstrated no significant concentration increase (+0.6 *μ*mol/L, *P* > 0.1). The distribution of 1,5-AH responses is shown in Figure 4(e). The majority of HE3286 patients significantly increased 1,5-AH, compared to placebos (15/18 versus 11/24, *P* = 0.02, Fisher's exact test). This outcome indicates that HE3286 had a treatment effect to decrease postprandial glucose excursions compared to placebo, which further supports that it's pharmacologic property is to decrease insulin resistance (see [9] and Figure 4(b)) and lower HbA1c.

### 3.10. HE3286-0401 Heteroscedasticity in HE3286 and Placebo Groups

Heteroscedasticity (differences in variances between groups) was investigated by analyzing data distributions for normality (Shapiro-Wilks *W* test) and analyzing dispersion (2-sided *F* test). In cohort 1 placebo, but not HE3286, day 84 distributions (*W* test) were significantly abnormal for changes in insulin, C-peptide, fasting glucose, HOMA2 %B, HOMA2 IR, and leptin in all subjects, and for changes in HbA1c, fasting glucose, and HOMA2 %B for MCP-1 > 40 pmol/L participants. Variances for cohort 1 placebo subjects (*F* test) were also significantly higher than those of HE3286 subjects for insulin, C-peptide, and HOMA2 IR for all subjects. 

Cohort 2 placebo, but not HE3286 distributions were abnormal (*W* test) for the group as a whole for changes in all the following parameters: day 84 nHbA1c, fasting glucose, MCP-1, and triglycerides and day 112 nHbA1c, fructosamine, and HOMA2 %B. In the BMI > 31 subgroup, abnormal distributions were found for changes in all the following parameters: day 84 HOMA2 %B and day 112 insulin, C-peptide, HOMA2 %B, and HOMA2 IR. Variances for cohort 2 placebo subjects as a whole were significantly higher (*F* test) for changes in all of the following parameters: day 84 insulin, HOMA2 %B, and triglycerides, and day 112 insulin and HOMA2 %B. Variances in placebo were also higher for the BMI > 31 kg/m^2^ subgroup for changes in day 84 MCP-1 and triglycerides and day 112 insulin (Table 5). 

These differences in distribution and dispersion between groups were not readily evident until day 84 of treatment (data not shown). Together, these findings further support an HE3286 treatment effect that decreases random metabolic effects and restores homeostasis to uncontrolled T2DM patients.

## 4. Discussion

### 4.1. Study HE3286-0401

This initial clinical trial of HE3286 in diabetes was designed to take all eligible patients with uncomplicated T2DM even though HE3286 was only qualified in animal models of obese diabetes and subsequently only demonstrated activity in obese individuals that present with inflammation-induced insulin resistance. The strategic intent of the study was to identify the responding T2DM population by surveying a broad swath of the constellation of syndromes that are defined by the T2DM condition. 

Based on findings in cohort 1, which indicated low BMI individuals were HE3286 nonresponders, and the inclusion criteria in the second cohort of the trial were modified, concentrating the population to elevated weight (BMI) and inflammatory status (MCP-1). Additional criteria included a requirement for detectable insulin and C-peptide levels. This eliminated the patient population that had progressed to lose significant *β*-cell function and who were no longer able to produce insulin, a population clearly not indicated for treatment with an insulin sensitizer. Notably, these criteria were also imposed on clinical trials with the thiazolidinediones (J. Olefsky, personal communication). In addition treatment-naïve patients were recruited in cohort 2 to remove the potential for metformin to blunt the HE3286 treatment effect and consequently amplify the single agent treatment outcome.

We designed this study to test the hypothesis, based on preclinical data and on molecular studies of HE3286 binding partners, that HE3286 would decrease the hyperactivation of NFkB with consequent restoration of insulin signaling [5, 6], dependent on its interaction with extracellular signal regulated kinase (ERK) 1 and 2 [7] in addition to other binding partners. ERK1 is an important mediator of inflammation-induced insulin resistance [23–25], insulin receptor substrate (IRS)-1 serine (inhibitory) phosphorylation, and the inhibitory effect of TNF*α* on insulin signaling [26]. HE3286 does not inhibit insulin-mediated ERK activation, but inhibits LPS- and TNF*α*-stimulated ERK hyper-activation, and IRS-1 serine phosphorylation mediated by IKK and JNK [5, 6]. Coincident HE3286-mediated changes in ERK, IKK, JNK, and p38 MAPK signal transduction may explain the preferential responses observed in high adiposity inflamed T2DM patients. Signal transduction pathways in omental fat are altered in obese, compared to lean individuals. In humans, activation of JNK and p38 MAPK was increased in omental fat (compared to paired subcutaneous fat) from obese, but not lean individuals, and this hyperphosphorylation correlated with clinical parameters of hyperglycemia and insulin resistance [27]. It will be important to further clarify the role of ERK in the activity of HE3286.

### 4.2. HE3286 Correlates

Data analysis presented here demonstrated that the cohort 1 day 84 changes in the primary end point HbA1c had a significant relationship with expected changes in beta-cell function, fasting glucose, and weight, and also with baseline inflammation status (MCP-1). Surprisingly a relationship with hemoglobin was also detected, a biomarker that is presumed stable over several weeks. Of these covariates associated with HbA1c change, only fasting glucose was significant in placebo patients.

In the enriched cohort 2 population, the HbA1c HE3286 treatment response was no longer dependent on MCP-1 but rather BMI with a statistically significant negative correlation; the higher the BMI the larger the effect on HbA1c decline. Higher BMI subjects presented with higher MCP-1. The cohort 2 outcome remained correlated with expected changes in *β*-cell function and with fasting glucose.

Thus the general population enrolled in cohort 1 was a very different ensemble of participants than those enrolled in cohort 2. While the relationships of change in HbA1c with changes in *β*-cell function and fasting glucose remained, the relationship to weight loss was not seen in the cohort 2 participants selected with higher BMI inclusion criteria. 

### 4.3. Placebo Correlates

Cohort 1 placebo group HbA1c change was dependent only on baseline inflammation status (TNF*α* and day 84 TNF*α* change). In cohort 2, there were no placebo correlates to HbA1c change. Importantly, fasting glucose change was not correlated with HbA1c change in this group, indicating that glucose levels were uncoupled from the HbA1c surrogate marker. Rather, placebo HbA1c variance (CV) was correlated strictly with inflammation in both cohorts. This was evidenced by correlation to baseline TNF*α* in cohort 1 and dependent on both changes in MCP-1 and surprisingly hemoglobin CV in cohort 2. Hb CV was in turn dependent on baseline MCP-1 (TNF*α* was not measured). 

In cohort 1 this later dependency on Hb CV was not detected perhaps due to the heterogeneity of the more general patient population (including nonobese and noninflamed diabetics). In cohort 2, placebo HbA1c CV was negatively correlated with weight change, indicating that higher weight led to increased variance. Cohort 2 placebo weight loss was unexpectedly unrelated to HbA1c and glucose control. Since placebo HbA1c CV was correlated with TNF*α* change in cohort 1, the weight loss associated with higher HbA1c CV in the cohort 2 placebo group is presumed to be related to inflammation effects on satiety or metabolism leading to changes in caloric intake and energy balance. 

### 4.4. HE3286 Treatment Effects

The significant correlation of changes in HbA1c and hemoglobin was an unexpected observation as hemoglobin is considered an invariant biomass from which HbA1c is formed as a reflection of total hyperglycemia and therefore its status as an FDA approved biomarker. Inspection of individual patient HbA1c changes revealed a high degree of intravisit variance, contrary to its presumed highly controlled and stable total body mass. Further exploration of the hematopoietic elements gathered with the safety data demonstrated these variant effects were not only on the hemoglobin mass but also on other components such as RBC, hematocrit, mean red cell volume, mean corpuscular hemoglobin and platelets, as well as a variety of metabolic parameters including glucose and cholesterol.

Unexpected variance in metabolic and hematologic parameters related to the effects of chronic low-grade inflammation in uncontrolled obese diabetes produced a significant barrier to these analyses and data interpretation. The variances that caused differing distributions and dispersions between treatment and placebo groups' coupled with the HE3286 treatment effect presented significant statistical challenges. Statistically random effects in the treatment-naïve placebo group were demonstrated for day 84 changes in glucose and in the key surrogate parameters hemoglobin, HbA1c and HOMA2 %B. The increasing variances in individual HbA1c change with metabolic disease progression demonstrated median changes of zero for dyslipidemic, IGT, and T2DM patients. Statistically, random effects are assumed to be the realization of a normal distribution with a mean of zero and a variance that can be estimated. In order to investigate HE3286 treatment effects, we were prompted to remove this random component by normalizing HbA1c to the day 84 average total hemoglobin mass (mean 84-day change of zero) for each patient. 

In the broadly defined population of metformin-treated T2DM patients (cohort 1), the HE3286 responsive patient population was found in the upper two tertiles of the inflammation marker MCP-1 (≥40 pmol/L). In the inflamed treatment-naïve patients' population studied in cohort 2, the responding population was found above the median BMI (obese subjects, >31 kg/m^2^). The magnitude of the treatment response was indeed greater in the treatment-naïve (cohort 2) than metformin-treated patients.

In both cohort 1 and 2, HE3286 treatment was associated with a total Hb mass normalization evidenced by day 84 data distributions and decreased variances in numerous metabolic and erythroid values. For several of these dysregulated parameters, HE3286 did not show a significant correction until day 84. We interpret these results to indicate that HE3286, via its anti-inflammatory activity, decreased inflammation-driven metabolic dysregulation. 

HE3286 showed a significant effect to improve insulin resistance in IGT subjects [9] and to decrease HOMA2 IR in cohort 1 T2DM patients, but not in cohort 2. It is possible that since cohort 2 was naïve, previously untreated T2DM patients and showed higher variances, additional improvements would be observed with longer treatment time frames or drug combination therapy. 

### 4.5. Metabolic Disease, Variance, and Random Effects

The relationship between inflammation and increased variances in erythroid and metabolic laboratory parameters was investigated in clinical settings of increasing chronic low-grade inflammation, adiposity, and metabolic dysregulation. Compared to a healthy group, significantly increased variances were observed for hematocrit, and HbA1c for dyslipidemic, IGT, and T2DM patients. RBC and hemoglobin values were also significantly variable, and the fasting glucose was variable in both dyslipidemic and T2DM patients. In treatment-naive T2DM, high variances and random effects were observed in a large number of metabolic and hematologic parameters that the medical community relies on for medical diagnoses. These changes were correlated with increased inflammatory mediators. This data supports our hypothesis that, in obese subjects, adipose tissue inflammation contributes to both metabolic and hematologic dysregulation within the same individuals. 

This is the first clinical report of extreme fluctuations in the marker HbA1c in patients with uncontrolled type 2 diabetes mellitus, but there are published data for type 1 diabetes mellitus (T1DM). Fluctuations in %HbA1c of more than 1% occurred in 50% of the patients year to year, and over 9 years the minimum-maximum range was >3% and >5% HbA1c in 55% and 11% of patients, respectively, [17]. In T1DM subjects followed for 4 years, there was high CV for intraindividual HbA1c measurements (15.5 ± 8.1%), which was lower for patients with good glycemic control. Intrasubject variations of fasting glucose and HbA1c (HbA1c 6–8%, with <10% variation in HbA1c over the last two months) were determined in healthy subjects and T1DM patients with good glycemic control [18]. Glucose intrasubject CV, were 5.4% (range 4.6–6.0) for healthy and 30.5% (26.7–35.5) for T1DM. HbA1c CV, were 1.2% (1.1–1.4) for healthy and 1.7% (1.5–1.9) for T1DM with good glycemic control. Longitudinal changes in T1DM glycemic control gave a significant positive association between baseline HbA1c and CV for intraindividual HbA1c (*P* < 0.01) [19].

HbA1c is a useful marker for detection of patients with elevated fasting and postprandial glucose. The ADA recommends that anyone with HbA1c >7 be treated with additional agents to return them to a glucose-controlled state. In this specific patient population of type 2 diabetes with HbA1c that is uncontrolled according to the ADA recommendations, the authors have found that the basic hypothesis of stable hemoglobin and red cell lifespan allowing extrapolation from HbA1c change and glucose control is flawed, that intravisit fluctuations can be large, and that a change in HbA1c values between two visits is unlikely to reflect a meaningful therapeutic effect on glucose control in this uncontrolled population. Thus, in clinical efficacy studies in patients with poorly controlled HbA1c, the authors recommend that additional tests of glucose control be used for determination of efficacy of new antidiabetic therapies. Numerous publications argue for the improved management by using continuous glucose monitoring, and for the time-averaged effects of using 1,5-anhydroglucitol [28] to better understand variation in glucose control. 

### 4.6. HE3286-0401 Conclusions

The hypotheses tested in this study appear to be borne out in the high adiposity T2DM patient. HE3286 preferentially improved clinical parameters in obese inflamed insulin-resistant T2DM patients. Since inflammatory changes were driving HbA1c changes in the placebo group for both cohorts 1 and 2, the changes observed with HE3286 treatment appear to be due to its anti-inflammatory activity (i.e., to break the cycle of inflammatory kinase-mediated inhibition of insulin receptor signaling). Furthermore, T2DM subjects that lack chronic, low-grade inflammation lack the specific lesion in the insulin receptor signaling pathway that HE3286 was developed to interdict. Their glucose intolerance arises for other reasons, and therefore they are unaffected by HE3286. 

Obese type 2 diabetic incidence is increasing at an alarming rate. Regaining glucose control and metabolic regulation and preventing or delaying macrovascular and microvascular complications could help to contain rising health care costs for end-stage diabetes complications. Understanding which patients are to benefit from a new therapy is now a regulatory consideration. The FDA has published the Critical Path Initiative, with personalized medicine, or the patient-specific information to individualize therapy and disease management as a major theme, and published on the importance of clinical validation of personalized medicine selection criteria in diabetes [29]. Based on estimates of obese, inflamed diabetics in the future at approximately 50% (J. Olefsky, personal communication), HE3286 offers a potentially important personalized medicine for these subjects. 

HE3286 is active at low (hormonal level) doses and is an anti-inflammatory insulin sensitizer with a toxicology profile conducive for chronic daily use [1]. In the responsive subpopulations HE3286 significantly decreased HbA1c compared to placebo, by day 84 in metformin-treated subjects with high MCP-1, and by day 112 in treatment-naïve subjects with high BMI. The data presented here in uncontrolled T2DM patients make a compelling argument for further testing of HE3286 in the high adiposity, inflamed T2DM patient subset, using oral glucose tolerance testing, 1,5-AH, or continuous glucose monitoring to assess treatment effects. The correlation or lack thereof with the surrogate marker HbA1c should be confirmed in these uncontrolled patients.

## Figures and Tables

**Table 1:** Correlations with individual treatment-naïve T2DM HbA1c and insulin coefficients of variation.

HbA1c CV^a^	*n*	Pearson correlation	*P* value
Hemoglobin CV	28	0.44	0.018
MCP-1 CV	20	0.44	0.0495
Glucose CV	28	0.58	0.0009
C-reactive protein CV	27	0.39	0.045
HDL CV	27	0.68	<0.0001
LDL CV	22	0.52	0.013
Triglyceride CV	28	0.62	0.0004
Lymphocyte CV	26	0.44	0.0261
Monocyte CV	27	0.56	0.0026
Platelet CV	28	0.42	0.025
Red blood cell CV	28	0.53	0.0036
Hematocrit CV	28	0.48	0.0094
MCV CV	28	0.38	0.048

Insulin CV	*n*	Pearson correlation	*P* value

Glucose CV	28	0.46	0.013
White blood cell CV	28	0.46	0.013
Neutrophil CV	28	0.54	0.0028

^a^HbA1c: hemoglobin A1c; CV: coefficient of variation; MCP-1: monocyte chemoattractant protein 1; HDL: high-density lipoprotein; LDL: low-density lipoprotein; MCV: mean corpuscular volume.

**Table 2:** HE3286-0401 baseline demographics and characteristics of each group.

Cohort 1	All Subjects		MCP-1^a^ > 40 pmol/L		MCP-1 ≤ 40 pmol/L	
	HE3286	Placebo	HE3286	Placebo	HE3286	Placebo
*n*	34	38	22	25	12	13

Age	48 (41–57)^b^	50 (43–59)	49 (40–57)	12(44–61)	48 (41–57)	48 (43–56)
Gender F (%)	19 (56%)	18 (47%)	12 (56%)	12 (48%)	7 (58%)	6 (46%)

Ethnicity						
White/White Hispanic	25 (74%)	27 (71%)	18 (82%)	18 (72%)	7 (58%)	9 (69%)
Black	5 (15%)	7 (18%)	2 (9%)	3 (12%)	3 (25%)	4 (31%)
Asian	3 (9%)	3 (8%)	1 (5%)	3 (12%)	1 (8%)	0 (0%)
Other	1 (3%)	1 (3%)	1 (5%)	1 (4%)	1 (8%)	0 (0%)

MCP-1 (pmol/L)	50 (35–87)	52 (34–70)	61 (50–75)	61 (52–81)	32 (27–36)	18 (20–36)
TNF*α* (pmol/L)	0 (0–0.1)	0 (0–0.3)	0 (0–0.1)	0 (0–0.4)	0 (0–0)	0 (0–0.4)
BMI (kg/m^2^)	29 (25–32)	30 (26–36)	29 (26–32)	29 (25–37)	27 (25–31)	33 (29–36)
HbA1c (%Hb)	8.5 (7.8–9.3)	8.3 (8.0–9.2)	9.0 (7.9–9.6)	8.3 (8.0–9.3)	8.0 (7.8–8.6)	8.5 (8.0–9.2)
Hb (mmol/L)	8.5 (8.1–9.3)	8.5 (8.1–9.3)	8.6 (8.1–9.3)	8.3 (8.1–9.3)	8.0 (7.5–8.7)	9.1 (8.1–9.3)
Hct (proportion)	0.41 (0.37–0.43)	0.41 (0.39–0.44)	0.41 (0.39–0.44)	0.40 (0.39–0.43)	0.39 (0.36–0.42)	0.44 (0.39–0.46)
RBC (10^12^/L)	4.6 (4.3–4.9)	4.7 (4.4–4.9)	4.6 (4.4–5.0)	4.7 (4.4–5.0)	4.6 (4.3–4.9)	4.7 (4.4–5.1)
Insulin (pmol/L)	56 (28–90)	63 (35–118)	69 (29–97)	56 (28–104)	56 (42–76)	90 (35–188)
C-peptide (nmol/L)	0.77 (0.60–1.0)	0.90 (0.53–1.2)	0.80 (0.50–1.1)	0.70 (0.53–1.1)	0.73 (0.67–0.87)	1.0 (0.53–1.4)
Fasting glucose (mmol/L)	8.9 (7.7–11)	9.2 (7.5–10.1)	9.5 (8.3–11)	8.4 (7.5–10)	8.1 (7.5–8.7)	9.2 (7.2–10)
1,5-Anhydroglucitol (*μ*mol/L)^c^	35 (12–60)	52 (20–85)				
CRP (pmol/L)	30 (14–61)	25 (12–73)	29 (12–49)	13 (11–73)	35 (18–94)	26 (15–57)
HOMA2 %B	47 (36–62)	59 (33–82)	41 (33–57)	58 (30–80)	58 (47–67)	70 (37–88)
HOMA2 IR	2.0 (1.5–2.7)	2.2 (1.4–3.2)	2.3 (1.3–2.9)	2.0 (1.4–2.9)	1.9 (1.6–2.3)	2.8 (1.3–3.6)
Triglycerides (mmol/L)	1.8 (1.3–2.9)	2.0 (1.3–2.5)	1.7 (1.2–3.1)	2.1 (1.3–2.7)	2.0 (1.4–2.6)	2.0 (1.3–2.1)

Cohort 2	All subjects		BMI > 31 kg/m^2^		BMI ≤ 31 kg/m^2^	
	HE3286	Placebo	HE3286	Placebo	HE3286	Placebo

*n*	25	28	12	15	13	13

Age	54 (48–60)	53 (46–58)	53 (42–56)	53 (46–55)	55 (49–63)	58 (46–62)
Gender F (%)	10 (40%)	14 (50%)	4 (33%)	9 (60%)	6 (46%)	5 (38%)

Ethnicity						
White/White Hispanic	20 (80%)	27 (96%)	10 (83%)	15 (100%)	10 (77%)	12 (92%)
Black	3 (12%)	0 (0%)	2 (17%)	0 (0%)	1 (8%)	0 (0%)
Asian	2 (8%)	1 (4%)	0 (0%)	0 (0%)	2 (15%)	1 (8%)
Other	0 (0%)	0 (0%)	0 (0%)	0 (0%)	0 (0%)	0 (0%)

BMI (kg/m^2^)	31 (29–33)	31 (29–37)	33 (32–36)	36 (32–37)	29 (28–30)	29 (28–30)
MCP-1 (pmol/L)^d^	108 (51–189)	94 (64–117)	97 (49–182)	94 (62–129)	110 (68–195)	95 (67–123)
HbA1c (%Hb)	8.1 (7.5–8.7)	8.5 (7.7–10.2)	7.6 (8.0–8.4)	8.4 (7.5–9.8)	8.4 (7.5–8.8)	9.4 (8.1–10.6)
Hb (mmol/L)	9.4 (8.7–9.9)	8.8 (8.1–9.9)	9.5 (8.7–9.9)	8.9 (8.1–9.9)	8.9 (8.7–9.9)	8.8 (8.1–9.9)
Hct (proportion)	0.45 (0.41–0.46)	0.42 (0.39–0.46)	0.45 (0.43–0.46)	0.44 (0.39–0.46)	0.42 (0.40–0.47)	0.42 (0.38–0.46)
RBC (10^12^/L)	4.7 (4.5–5.1)	4.7 (4.5–5.1)	4.9 (4.6–5.1)	4.8 (4.5–5.0)	4.7 (4.3–5.0)	4.5 (4.3–5.1)
Insulin (pmol/L)	104 (69–146)	111 (76–153)	118 (76–139)	118 (76–146)	97 (69–153)	104 (69–174)
C-peptide (nmol/L)	1.1 (0.8–1.3)	1.1 (0.9–1.4)	1.2 (0.8–1.3)	1.2 (1.0–1.3)	1.0 (0.8–1.2)	1.0 (0.9–1.5)
Fasting glucose (mmol/L)	8.3 (7.2–9.9)	9.2 (7.3–11)	8.0 (6.7–8.8)	8.2 (6.8–10)	8.5 (7.2–11)	9.3 (8.1–13)
1,5-Anhydroglucitol (*μ*mol/L)^e^	54 (31–83)	102 (35–154)				
CRP (pmol/L)	27 (19–49)	32 (16–48)	27 (22–67)	33 (22–53)	28 (13–48)	30 (12–45)
HOMA2 %B	74 (43–108)	61 (45–89)	87 (47–123)	80 (43–120)	71 (35–85)	56 (46–73)
HOMA2 IR	2.7 (2.2–3.4)	2.8 (2.5–3.8)	2.9 (2.1–3.4)	3.2 (2.5–3.9)	2.6 (2.2–3.3)	2.7 (2.5–4.3)
Triglycerides (mmol/L)	1.6 (1.1–2.7)	2.1 (1.5–3.5)	1.4 (1.1–1.8)	2.0 (1.6–2.8)	1.4 (2.3–3.2)	3.5 (1.4–6.7)

^a^MCP-1: monocyte chemoattractant protein 1; TNF*α*: tumor necrosis factor alpha; BMI: body mass index; HbA1c: hemoglobin A1c; Hb: hemoglobin; Hct: hematocrit; RBC: red blood cells; CRP: C-reactive protein; HOMA2 %B: homeostatic model assessment of % pancreatic beta cell function, HOMA2 IR: homeostatic model assessment of insulin resistance.^ b^Numbers are medians (IQR) or numbers (%). ^c^1,5-Anhydroglucitol only 19 retention samples (9 HE3286, 10 placebo, predominantly with MCP > 40 pmol/mL). ^d^MCP-1 only 38 samples (18 HE3286, 20 placebo); ^e^1,5-Anhydroglucitol only 23 retention samples (9 HE3286, 14 placebo, predominantly from BMI > 31 kg/m^2^ subjects).

**Table 3:** HE3286-0401 significant correlates of HbA1c and Hb changes.

Group	Dependent	Independent	Test	HE3286				Placebo			
				*n *	*r *	95% CI	*P *	*n *	*r *	95% CI	*P *
Cohort 1	ΔHbA1c^a^	MCP-1	Spearman	34	−0.42	−0.67 to −0.08	**0.01**				>0.10
		ΔHOMA2 %B	Spearman	34	−0.43	−0.68 to 0.10	**0.01**				>0.10
		ΔGlucose	Spearman	34	0.45	0.11 to 0.69	**0.009**				>0.10
		ΔWeight	Spearman	34	0.45	0.13 to 0.69	**0.007**				>0.10
		ΔHb	Pearson	34	0.36	0.03 to 0.62	**0.03**				>0.10
	ΔHbA1c	TNF*α*	Spearman				>0.10	34	−0.48	−0.71 to −0.16	**0.004**
		ΔGlucose	Spearman				>0.10	38	0.65	0.41 to 0.81	**<0.0001**
		ΔTNF*α*	Spearman				>0.10	32	0.40	0.05 to 0.66	**0.02**
	HbA1c CV	TNF*α*	Pearson				>0.10	34	0.50	0.19 to 0.72	**0.002**

Cohort 2	ΔHbA1c	BMI	Spearman	25	−0.41	−0.70 to −0.008	**0.04**				>0.10
		ΔHOMA2 %B	Pearson	25	−0.50	−0.75 to −0.13	**0.02**				>0.10
		ΔGlucose	Pearson	25	0.61	0.29 to 0.81	**0.001**				>0.10
	HbA1c CV	Hb CV	Pearson				>0.10	28	0.44	0.08 to 0.70	**0.02**
		ΔMCP-1	Pearson				>0.10	20^b^	0.44	0.002 to 0.74	**0.0495**
		ΔWeight	Pearson				>0.10	28	−0.43	−0.69 to −0.07	**0.02**
	Hb CV	MCP-1	Spearman				>0.10	20	0.38	0.03 to 0.70	**0.04**

Cohort 2 BMI > 31	ΔnHbA1c	MCP-1	Spearman Exact	10^b^	−0.68	—^c^	**0.03**				>0.10
		ΔMCP-1	Spearman Exact	9^b^	0.77	—	**0.002**				>0.10

^a^Δ: change in; CV: coefficient of variation; HbA1c: hemoglobin A1c; Hb: hemoglobin; MCP-1: monocyte chemoattractant protein-1; HOMA2 %B: homeostatic model assessment of pancreatic beta cell function; TNF*α*: tumor necrosis factor alpha; BMI: body mass index; nHbA1c: HbA1c normalized to day 84 hemoglobin mass (see Section 2 for details). ^b^MCP-1 data unavailable on a portion of participants. ^c^Spearman Exact test has no confidence interval.

**Table 4:** HE3286-0401 treatment effects in obese inflamed subgroups.

Group	Effect	Value	Change		*P *	Test^g^
			HE3286	Placebo		
Cohort 1 MCP > 40^a^	ΔHOMA2 IR^c^	Day 84 mean	−0.1	+0.4	**0.02**	
	ΔC-peptide		−0.03	+0.1	**0.04**	
	ΔHb		−0.25	+0.06	**0.02**	*t*-test
	ΔHct		−0.06	+0.09	**0.02**	
	ΔRBC		−0.05	+0.09	**0.02**	
	ΔnHbA1c	Day 84 median	−0.34		**0.03**	Wilcoxon
		Day 84 median		0.1	—	
		Day 84 numbers	17⇓^e^ 5⇑^f^	9⇓ 16⇑	**0.0008**	Fisher's Exact
		Day 84 mean	−0.46	−0.21	—	
		Day 84 mean −2 outliers^d ^	−0.82	−0.21	**0.04**	*t*-test

Cohort 2 BMI > 31^b^	ΔnHbA1c	Day 112 mean	−1.0		**0.0007**	
				−0.3	—	*t*-test
			−1.0	−0.3	**0.03**	
		Day 112 median	−1.2		**0.002**	Wilcoxon
				−0.16	—	
			−1.2	−0.16	**0.02**	Mann Whitney

^a^Participants with baseline monocyte chemoattractant protein greater than the lowest tertile (40 pmol/L). ^b^Participants with baseline body mass index greater than the median (31 kg/m^2^). ^c^Δ: change in; HOMA2 IR: homeostatic assessment model insulin resistance; Hb: hemoglobin; Hct: hematocrit, RBC: red blood cells; nHbA1c: normalized HbA1c (see Section 2 for details); ^d^Two outliers removed (outliers circled in Figure 4(c), Mahalanobis distance); ^e^Decrease from zero change; ^f^Increase from zero change; ^g^Parametric means and *t*-test used for data with normally distributed data, Nonparametric medians, Wilcoxon, Mann Whitney, and Fisher's Exact test used for abnormally distributed data.

**Table 5:** HE3286-0401 Heteroscedasticity^a^ Between HE3286 and Placebo Changes from Baseline Values in Laboratory Parameters.

Group	Day	Parameter	HE3286	HE3286 > Placebo		Placebo	Placebo > HE3286
			*W* test *P *		*F* test *P *	*W* test *P *	*F* test *P *
Cohort 1	84	ΔInsulin^d^	>0.1		>0.1	**<0.0001**	**0.007**
		ΔC-peptide	>0.1		>0.1	**<0.0001**	**0.0495**
		ΔFasting glucose	>0.1		>0.1	**0.02**	>0.1
		ΔHOMA2 %B	>0.1		>0.1	**<0.0001**	>0.1
		ΔHOMA2 IR	>0.1		>0.1	**0.002**	**0.049**
		Δleptin	>0.1		>0.1	**0.005**	>0.1
Cohort 1 MCP-1 > 40^b^	84	ΔHbA1c	>0.1		>0.1	**0.006**	>0.1
		ΔFasting glucose	>0.1		>0.1	**0.02**	>0.1
		ΔHOMA2 %B	>0.1		>0.1	**<0.0001**	>0.1

Cohort 2	84	ΔnHbA1c	>0.1		>0.1	**0.04**	>0.1
		ΔInsulin	>0.1		>0.1	>0.1	**0.004**
		ΔFasting glucose	>0.1		>0.1	**0.03**	>0.1
		ΔHOMA2 %B	>0.1		>0.1	>0.1	**0.006**
		ΔMCP-1	>0.1		>0.1	**0.005**	>0.1
		ΔTriglycerides	>0.1		>0.1	**<0.0001**	**0.007**
	112	ΔnHbA1c	>0.1		>0.1	**0.0007**	>0.1
		ΔInsulin	>0.1		>0.1	>0.1	**0.008**
		ΔFructosamine	>0.1		>0.1	**0.002**	>0.1
		ΔHOMA2 %B	>0.1		>0.1	**<0.0001**	**0.01**

Cohort 2 BMI > 31^c^	84	ΔHOMA2 %B	>0.1		>0.1	**0.007**	>0.1
		ΔMCP-1	>0.1		>0.1	>0.1	**0.009**
		ΔTriglycerides	>0.1		>0.1	>0.1	**0.001**
	112	ΔInsulin	>0.1		>0.1	**<0.0001**	**0.001**
		ΔC-peptide	>0.1		>0.1	**<0.0001**	>0.1
		ΔHOMA2 %B	>0.1		>0.1	**<0.0001**	>0.1
		ΔHOMA2 IR	>0.1		>0.1	**<0.0001**	>0.1
			>0.1		>0.1	>0.1	>0.1

^a^Heteroscedasticity describes differences in variances between groups.^ b^Participants with baseline monocyte chemoattractant protein greater than the lowest tertile (40 pmol/L, see results). ^c^Participants with baseline body mass index greater than the median (31 kg/m^2^, see results). ^d^Abbreviations: Δ: change in; HOMA2 %B: homeostatic model assessment of pancreatic beta cell function; HOMA2 IR: homeostatic model assessment of insulin resistance; HbA1c: hemoglobin A1c; nHbA1c: HbA1c normalized to 84 day average hemoglobin mass; MCP-1: monocyte chemoattractant protein-1.
